# Notes and News

**Published:** 1898-09-24

**Authors:** 


### Notes and News.

(Collected and Communicated.)

Yellow fever is reported to be raging in all the ports on the Mexican coast.

The in and out-patient departments of the Middlesex Hospital were re-opened on Monday, September 19th, for the reception of patients.

The Lord Bishop of Rochester will deliver the prizes to the students of St. Thomas's Hospital Medical hool at the hospital on Monday, October 3rd; at 3 p.m.

The introductory address to be given at the opening of the present session at the Medical School of St. Mary's Hospital will be delivered on Monday, October 3rd, at three o'clock, by Henry Caley, Esq., M.D.

The useful service rendered by the branch of the St. John Ambulance Association at the recent railway disaster has been much commended, and the Midland Railway Company has sent £20 to the corps in recognition of their valuable assistance.

At a recent quarterly court of the Governors of the London Hospital it was announced that, of the £100,000 appealed for, £77,000 in donations and £11,000 in annual subscriptions had been received. The Press Bazaar, as has been announced, contributed £12,000.

The Charity Organisation Society, the Society for the Prevention of Cruelty to Children, St. Mary's Hospital (Paddington), and the Royal Sea Bathing Infirmary (Margate) have each been left the handsome bequest of £5,000 under the will of the late Major-General Johnstone.

The Prussian Minister of the Interior has ordered that in future female medical practitioners are to be appointed for the examination of prostitutes brought in for the first time. This is distinctly a step in the right direction, and ought to lessen the objections raised in some quarters to all the attempts which have been made to place this dangerous trade under sanitary supervision.

The winter session of the Middlesex Hospital Medical School will commence on Monday, October 3rd, when an address will be delivered by Dr. Arthur Francis Voelcker at 3 p.m., after which the prizes gained by the students during the past session will be distributed by Sir Elliott Lees, Bart., M.P., and a banquet will be held in the evening at the Cafe Royal, under the presidenc of the Right Hon. Sir Ralph Thompson, K.C.B., Chairman of the Council of the Medical School.

It is curious to find that in the annual report of the Cincinnati Department of Health deaths from surgical operations are returned among the "Deaths from violence."

The Local Government Board have informed the Metropolitan Asylums Board that they have directed their chief officer, Mr. W. E. Knollys, C.B., to hold, after the vacation, an inquiry into the causes of the excess of cost over the tenders received for the erection, &c., of the Brook Hospital, the architect's responsibility, and the supervision exercised by the committee and the managers as regarded expenditure.

Plymouth ratepayers are not overpleased at the prospect of an expenditure of £50,000 to secure them a new hospital in connexion with their workhouse. The present building will receive 184 patients, but this accommodation is held to be inadequate, and the building generally not up to modern requirements. The present proposal provides for 350 beds. The building would be erected in handsome blocks replete with every modern convenience. The nurses' home would be detached from the main building, and a porter's lodge and other adjuncts form part of the scheme.

A very careful paper, by Dr. F. A. Dixey, on "Diphtheria in London, 1896-98," in which the subject of the incidence of the disease is treated statistically, shows that although the amount of diphtheria in London is still high, it is now diminishing after the temporary increase of 1896. It also brings forward fresh evidence to show that school infection is an important, although not the sole, cause of the spread of the disease, an interruption to the usual course of diphtheria mortality, coincident with the two chief school holiday periods, being again apparent both in 1896 and 1897 as it has been in previous years.

The problem set to the good people of Belfast by the present outbreak of typhoid fever seems to be a much more serious one than the mere rectification of an infected water supply would be. The wide-spread nature of the attack and its apparent want of exclusive association with either source of water supply, together with the ominous fact that the present epidemic, as it is called, is but an exacerbation of a general prevalence of fever which has been going on for a long time, all point to the fever being endemic in the city and to its being the result of a general fouling of the soil on which the houses are built, which it may take a long time and a large expenditure of money to put to rights.

The death of a medical man from accidental poisoning is reported from Great Budworth, in Cheshire. Dr. W. Howard Clark, the gentleman in question, was doing the work of Dr. Love, who was absent on his holiday. On returning from his rounds, Dr. Clark appeared greatly exhausted by the heat, and it is supposed that he at once took a tonic. At any rate, while at his dinner he complained of great pain, and cried out, "I am cramped all over!" and in a few minutes later he died. Subsequently it was discovered that the strychnine had been removed from the poison cupboard and stood on the surgery table, and an analysis of the dregs of a phial revealed a mixture of strychnine, ammonia, and gentian, which he had been known to take as a pick-me-up. From half to three-quarters of a grain of strychnine was found in the stomach. He had been in the best of health and spirits, and no doubt he accidentally took an overdose. Poor fellow!

